# Hemispheric asymmetry of the dayside aurora due to imbalanced solar insolation

**DOI:** 10.1038/s41598-020-70018-w

**Published:** 2020-08-10

**Authors:** Kan Liou, Elizabeth J. Mitchell

**Affiliations:** The Johns Hopkins University Applied Physics Laboratory, Space Exploration Sector, 11100 Johns Hopkins Road, Laurel, MD 20723 USA

**Keywords:** Space physics, Physics

## Abstract

Unlike the nightside aurora, which is controlled mainly by magnetic field reconnection in the magnetotail, the dayside aurora is closely associated with magnetic field merging at the dayside magnetopause. About two decades ago, it was discovered that the aurora is also controlled by solar insolation. Because the finding was based on data acquired mainly in the Northern Hemisphere, an outstanding question is if the auroral solar insolation effect also exists in the Southern Hemisphere. The present study addresses this question by studying dayside auroras from both hemispheres. We analyze 6 years’ worth of Earth disk emissions at far ultraviolet wavelengths acquired by the Global UltraViolet Imager on-board the Thermosphere Ionosphere Mesosphere Energetics and Dynamics satellite from 2002 to 2007. It is found that the solar insolation effect also exists in the Southern Hemisphere. In essence, the energy flux deposited as electron precipitation, is larger when the polar hemisphere is sunlit and is smaller when the polar hemisphere is dark. Because auroras are produced mainly by electron precipitation and because electrons are the main current carrier, this north–south asymmetry is consistent with the previous finding that larger (smaller) field-aligned currents are flowing out of the sunlit (dark) hemisphere. This trend is independent of the solar wind driving, suggesting that it is an effect associated with solar insolation. A small north–south asymmetry in the dayside auroral energy flux was identified. We discuss the asymmetry in the context of magnetospheric current and voltage generators.

## Introduction

The dayside aurora is loosely defined as auroral activity that occurs in the sunward half of the auroral oval delineated from the nightside aurora by the dawn-dusk meridian. When viewed from space in far ultraviolet (FUV), there is a distinctive difference between dayside and night auroras. Unlike the nightside aurora, which is produced mainly by particle precipitation originating from the nightside plasma sheet, the dayside aurora has its precipitating sources from a variety of magnetospheric regimes such as the cusp, mantle, lower-latitude boundary layer (LLBL), and the dayside extension of the central plasma sheet/boundary plasma sheets (CPS/BPS)^1^. Dayside auroras are most intense in the region from 1,400 to 1,600 magnetic local time (MLT)^2–4^, which is often referred to as the postnoon (or 1,500 MLT) auroral bright spot. The location of the peak intensity of the postnoon aurora coincides with the peak occurrence frequency of electron precipitation^5^. While precipitating particles that produce dayside auroras can have their magnetospheric origins from various magnetospheric source regions, there is evidence that the brightest auroral features seen in the postnoon sector are associated with particle precipitation from the BPS and near the boundary between the plasma sheet and other regions^3^.

Past studies have suggested that the intensity of dayside auroras is controlled by plasma and field parameters in the solar wind^6,7^. While the dayside aurora is closely associated with the solar wind-magnetosphere coupling, its intensity also depends on whether or not the local ionosphere is in sunlight. Using four months’ worth of FUV auroral images from Polar, it was shown, for the first time, that the intensity of postnoon auroras increase from April to July (e.g., increasing with the amount of sunlight received by the ionosphere)^8^. This finding was further demonstrated with significantly more data in a later study that showed dayside auroras are more intense in summer and less intense in winter^9^. This dayside auroral-sunlight effect was further confirmed with a different data set that covered ~ 6 years’ (2002–2007) worth of FUV auroral images acquired from the Global Ultraviolet Imager (GUVI) on board NASA's Thermosphere, Ionosphere, Mesosphere Energetics and Dynamics (TIMED) satellite^10^. In this work, it was found that dayside auroral enhancements are associated with electron precipitation with weaker energy flux (< ~ 5 erg/cm^2^-s) and average energy (< ~ 5 keV). They also found that suppression of the nightside aurora in sunlight was associated with larger energy flux (> ~ 10 erg/cm^2^-s) and average energy (> 10 keV). These findings are consistent with in situ particle measurements^11,12^.

The auroral solar insolation effect has been studied using auroral images acquired from the Northern Hemisphere^8,9^ or in situ particle data from combined hemispheric measurements^11,13^. It is still not known if such an effect exists in the Southern Hemisphere. Some scientists invoke a voltage generator in the magnetosphere to explain the auroral solar insolation effect on the dayside aurora^9^. A magnetospheric voltage generator would predict a symmetric response of auroral activity if the ionospheric conductance from the two hemispheres is identical. In this study we will focus on the following two questions: (1) If the well-known auroral-sunlight response that has been reported for the Northern Hemisphere is also present in the Southern Hemisphere and (2) if there is a difference in their response to sunlight?

## Data and analysis

**Figure 1:**
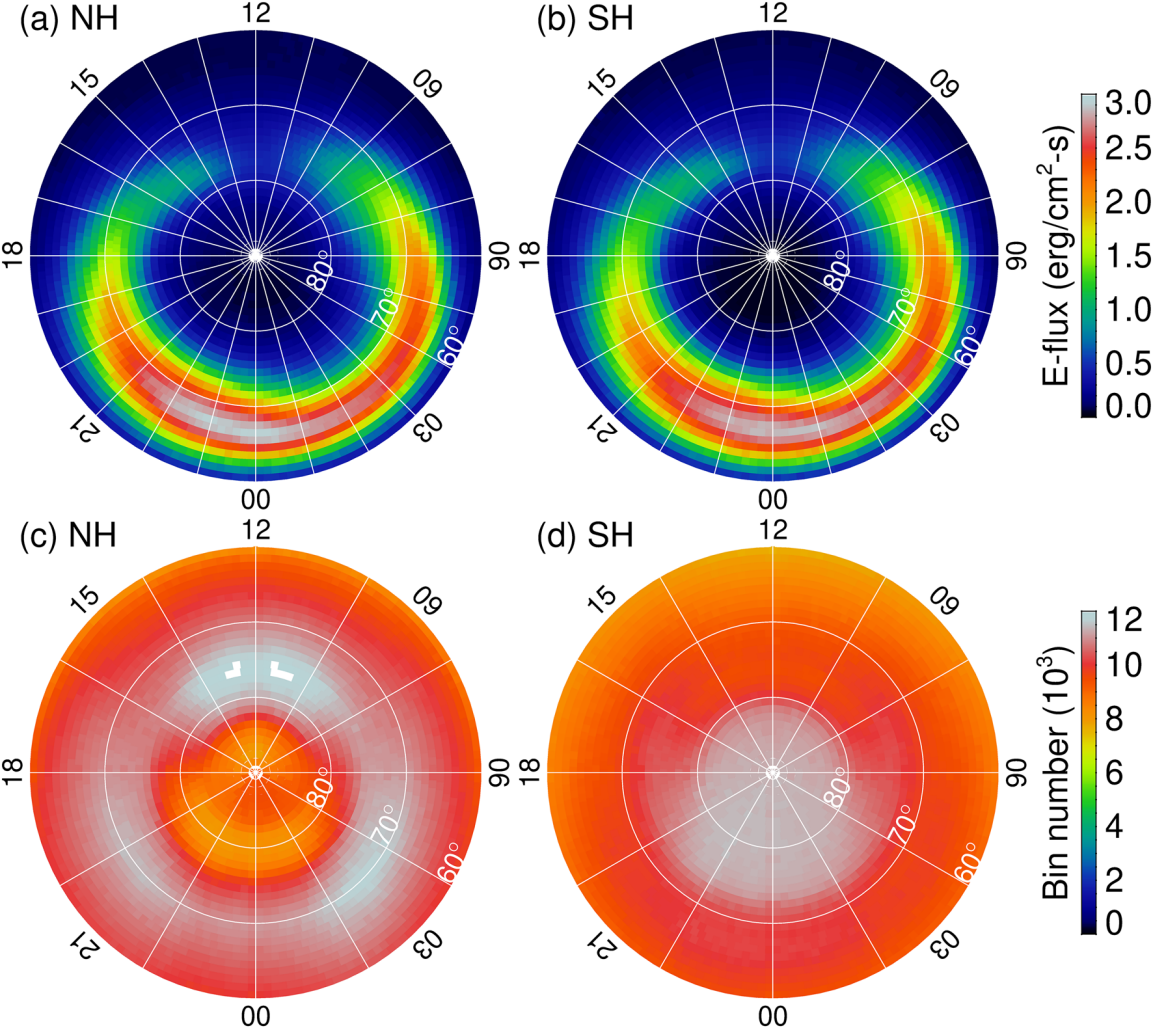
Longterm (2002–2007) averages of auroral energy flux inferred by TIMED/GUVI FUV auroral emission data for (**a**) Northern Hemisphere (NH) and (**b**) Southern Hemisphere (SH) in magnetic latitude (MLat)—magnetic local time (MLT) formats. The number of measurements per bin that are used to calculate the averaged energy is provided in (**c**) for NH and (**d**) for SH. There are 2,763 bins in total in each hemisphere. The figure is produced by the first author using the Interactive Data Language (IDL) software version 8.2.3. The ULR link for IDL is https://www.harrisgeospatial.com/.

It is desirable to compare snapshots of auroral images simultaneously from identical sensors and from the two opposite hemispheres. Unfortunately, such a data set does not exist, and a statistical study is the only way to reveal the auroral solar insolation effect. In this study we will analyze auroral images acquired by the Global Ultraviolet Imager (GUVI)^14^ aboard the TIMED spacecraft from February 2002 to November 2007. GUVI is a (mirror) scanning spectrometer in the far ultraviolet (FUV) wavelength^15^. The FUV emissions of the Earth’s disk are scanned horizon-to-horizon along the TIMED orbit, including the polar region. Each cross-track scan covers ~ 22.5° of longitudes (~ 2,500 km). Due to the “low” inclination (74°) and 625 km near-circular orbit, TIMED precesses ~ 3° per day and covers the entire local time in ~ 3 months. The present analysis and results comparison will be statistical because the TIMED single satellite observation cannot provide simultaneous snapshot auroras from both hemispheres. From 2002 to 2007, the total number of auroral oval images is 28,774 from the Northern Hemisphere and 29,742 from the Southern Hemisphere. It is desirable to present the auroral intensity in a more physical meaningful quantity for easy comparisons with other data. Therefore, we will analyze the inferred energy flux of electron precipitation from the Lyman–Birge–Hopfield long band within the GUVI FUV spectra^16^. We resample the energy flux data into 2,763 nearly uniform square bins, starting from 60° magnetic latitude (MLat) and magnetic midnight counting anti-clockwise from the magnetic midnight with 1° in latitude and an equal length in longitude, in the Altitude Adjusted Corrected Geomagnetic Magnetic (AACGM) coordinates^17^. Figure 1 shows a comparison of the statistical pattern of auroral energy flux from the two hemispheres. The number of measurements per bin for each hemisphere is also provided (lower panels) and is estimated to be on the order of ~ 10^4^ in the auroral region. This number is smaller than the number of the actual polar crossing by the TIMED spacecraft because each polar scan only covers no more than ~ 1/2 of the oval. As shown in the top panels, the energy flux patterns from the two hemispheres are nearly identical. The statistical energy flux resembles FUV auroral oval with a midday gap and a midnight maximum [e.g.,^8^]. A pixel-by-pixel linear regression analysis of the relationship between the northern and southern hemispheric energy flux gives the y-intercept equal to 0.014 ± 0.002 and the slope equal to 0.992 ± 0.002 (not shown), which is very close to one. The Pearson correlation coefficient is also close to one (*r* = 0.996). It is suggested that the long-term averages of the auroral energy deposited into the northern and southern hemispheres are nearly symmetric and identical.

Because there is a close relationship between dayside auroral particle precipitation and solar wind driving [e.g.,^6,18^], the effect of solar wind driving needs to be considered and controlled when studying the auroral sunlight effect. Here we simplify the solar wind driving by the solar wind magnetosphere coupling function, *dϕ*_*MP*_/*dt* (= C *v*^4/3^*B*_*t*_^2/3^*sin*^8/3^(*θ*_*c*_/2), where *v* is the solar wind speed, *B*_*t*_ is the component of the IMF transverse to the Sun–Earth line, and *θ*_*c*_ is the IMF clock angle)^19^, and use it to constrain the data. The solar wind magnetosphere coupling function is a proxy of the time rate of change of open magnetic flux (or merging rate) at the magnetopause. The coupling function can be converted to rate of opening of magnetic flux in Weber (Wb/s) by substituting 100 MWb nT^−4/3^ (km/s)^−2/3^ for C^20^. Typically, the value of *dϕ*_*MP*_/*dt* is under 2 MWb/s, with a median value of ~ 0.5 MWb/s. During geomagnetic active periods, such as storms and large substorms, nightside auroral activity can expand to dayside. We also calculate and use the time-weighted coupling function, 〈*dϕ*_*MP*_/*dt*〉, which is derived from the four previous hours of solar wind and IMF data^19^, to limit the nightside effect. The solar wind data are obtained from the 1-min OMNI data, which is time shifted to the subsolar bow shock^21^.

**Figure 2:**
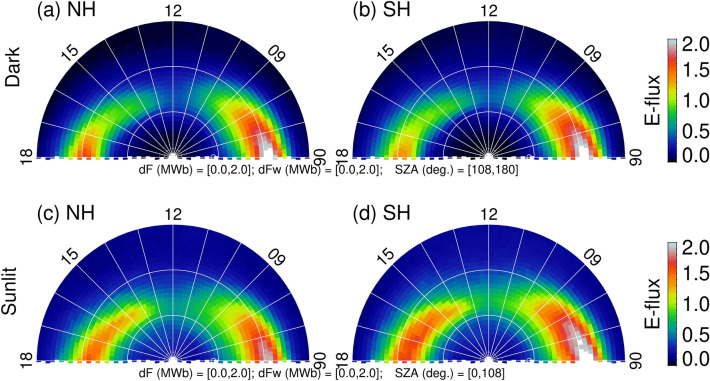
Averaged dayside auroral particle energy flux under (**a**,**b**) dark and (**c**,**d**) sunlit conditions for all solar wind driving (*dϕ*_*MP*_*/dt* = 0–2.0 MWb/s and 〈*dϕ*_*MP*_/*dt*〉 = 0–2.0 MWb/s). The figure is produced by the first author using the Interactive Data Language (IDL) software version 8.2.3. The ULR link for IDL is https://www.harrisgeospatial.com/.

To explore sunlight effects on northern and southern dayside auroras, we separate data using solar zenith angles (SZA; the angle between the zenith and the center of the Sun). Figure 2 shows dayside (06–18 MLT) auroral energy flux under dark (top panels) and sunlit (bottom panels) conditions. The solar wind driving, both *dϕ*_*MP*_/*dt* and 〈*dϕ*_*MP*_/*dt*〉, is set to a range between 0 and 2 MWb/s to exclude extreme events. We only present the dayside portion of the polar region poleward of 60° MLat. It would be misleading to show both dayside and nightside auroras together for the present study because dayside and nightside auroral activities are associated with different processes and have different response times to the solar wind driving. Furthermore, instead of binning data using summer and winter, we organize the data by sunlight. In the present study, we use the astronomical dawn (SZA = 108°, which corresponds to an ionospheric shadow height of ~ 327 km) to determine dark and sunlit conditions. That means pixels with SZA > 108° are considered in darkness and pixels with SZA < 108° are considered in sunlight. As shown in Fig. 2, there is a clear difference between the postnoon (around 14–16 MLT) energy flux, which is larger under sunlit than under dark ionospheric conditions, irrespective of the hemispheres. Moreover, the oval tends to be narrower in darkness and wider in sunlight. This appears in both hemispheres. The enhancement is more pronounced in the Southern Hemisphere. In the prenoon sector, changes in the energy flux exist for both hemispheres but not as significant. Previous studies have suggested that auroras in the prenoon sector behave like nightside auroras in response to nightside geomagnetic activity. This is because convecting magnetospheric electrons drift from the nightside to the dayside through the dawn flank. They precipitate mostly there, along the dawn flank, and rarely cross the noon meridian^1^.

**Figure 3:**
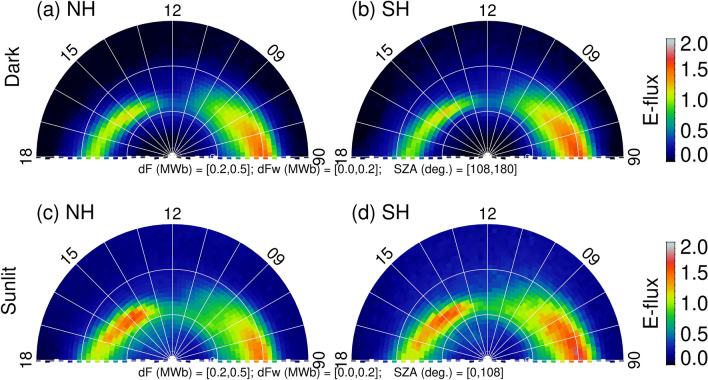
Averaged dayside auroral particle energy flux under (**a**,**b**) dark and (**c**,**d**) sunlit conditions for all IMF *B*_*y*_ and weak solar wind driving (*dϕ*_*MP*_*/dt* = 0.2–0.5 MWb/s and 〈*dϕ*_*MP*_/*dt*〉 = 0–0.2 MWb/s). The figure is produced by the first author using the Interactive Data Language (IDL) software version 8.2.3. The ULR link for IDL is https://www.harrisgeospatial.com/.

It has been shown that the brightest dayside aurora occurs statistically in the postnoon sector around 1,400–1,600 MLT^8^. However, there is clear evidence of an extension of the nightside aurora into the day sector in Fig. 2. That means nightside auroral activity may contribute to the average pattern. To minimize the nightside effect, the time-averaged solar wind driving is reduced to 0.0 < 〈*dϕ*_*MP*_/*dt*〉 < 0.2 MWb/s. In addition, the solar wind driving is limited to moderate values, e.g., 0.2 < *dϕ*_*MP*_/*dt* < 0.5 MWb/s. The result is shown in Fig. 3. Comparing Fig. 2 (all solar wind driving) and Fig. 3 (weak solar wind driving), there are a few significant differences in the postnoon aurora. First, the “auroral oval” is wider in latitude for all solar wind driving and is narrower for weak solar wind driving. This is not unexpected because a larger (smaller) variability in the solar wind control parameters would result in a larger (smaller) auroral variability. The auroral oval moves equatorward for all solar wind driving and poleward for small solar wind driving. This is also expected because the polar cap contracts when the solar wind driving weakens. Second, a local peak appears in the energy flux at ~ 1,500 MLT for weak solar wind driving. This is probably due to the imposed weak solar wind driving as a preceding solar wind condition. Under such a condition, nightside auroras would have retreated from dayside after the nightside geomagnetic activity has subsided. A distinct postnoon auroral form in the NH during weak solar wind driving has been shown with the Polar data^7^. Third, although the peak auroral energy flux in the 1,400–1,600 MLT sector is larger under weak solar wind driving than under all solar wind driving, the total energy flux is smaller under weak than under all solar wind driving. The latitudinally averaged energy flux over the local time sector from 60° to 85° MLat for NS and SH is 0.39 and 0.40 erg/cm^2^-s, respectively, for all solar wind driving and 0.35 and 0.34 erg/cm^2^-s, respectively, for weak solar wind driving.

**Figure 4:**
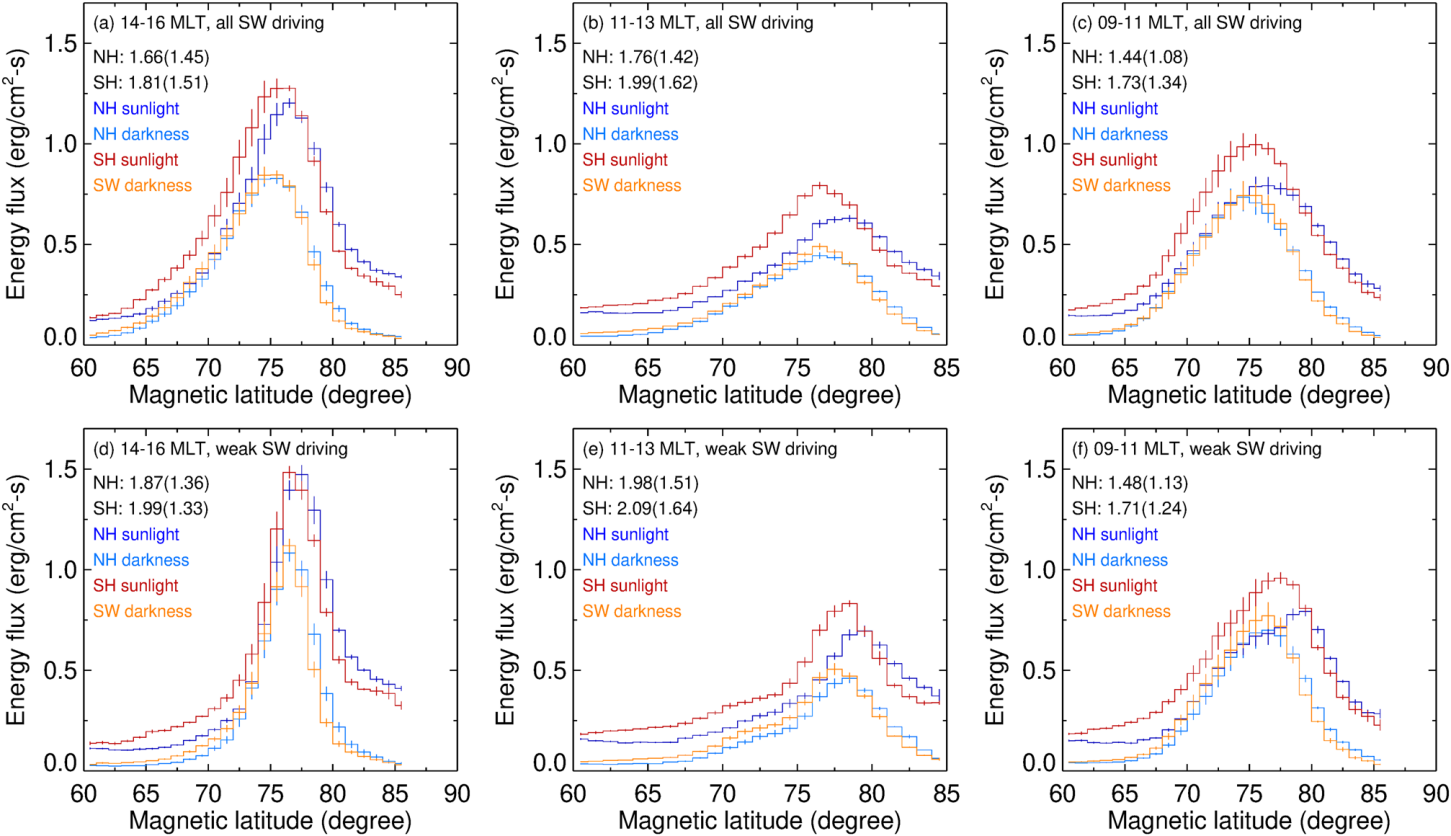
Comparisons of the latitudinal energy flux profile at (**a**,**d**) the 1,400–1,600 MLT, (**b**,**e**) 1,100–1,300 MLT, and (**c**,**f**) 0,900–1,100 MLT sectors for (top panels) all solar wind corresponding to Fig. 2 and (bottom panels) weak solar wind driving corresponding to Fig. 3. In each panel, blue is for sunlit NH, orange is for dark NH, red is for sunlit SH, and cyan is for dark SH. The vertical bar at each latitudinal bin represents standard deviation of the mean. The inserts in each panel are the sunlit-to-dark ratio of latitudinal integrated and peak (in the parenthesis) energy flux.

To provide quantitative measures of the dayside auroral enhancement from a dark to a sunlit ionosphere, we plot the latitudinal profile of averaged energy flux at three magnetic local time sectors in Fig. 4. The three local time sectors represent the statistical locations for the postnoon auroral hot spot (a and d: 1,400–1,600 MLT), midday aurora (b and e: 1,100–1,300 MLT), and the morning warm spot (c and f: 0,900–1,100 MLT). In general, there is a latitudinal difference in the auroral energy flux increase from dark to sunlit ionosphere. The sunlit-to-dark energy flux ratio increases roughly linearly with the latitude, from ~ 2 at 60° MLat to ~ 4 at 85° MLat. This trend appears in both hemispheres.

We also calculate the ratio of the total auroral energy flux (e.g., integrated over the sector) for sunlit and dark conditions and the result is provided as inserts in each panel. Regardless of the three local time sectors and the two hemispheres, the auroral energy flux is larger when the ionosphere is in sunlight (red and blue) than when the ionosphere is in darkness (orange and cyan). For example, the sunlit-to-dark ratio of the total energy flux within the 1,400 and 1,600 MLT sector is 1.66 and 1.81 for the NH and the SH, respectively, and the sunlit-to-dark ratio of the peak energy flux is 1.45 and 1.51 for the NH and the SH, respectively (see Fig. 4a). In addition, the sunlit-to-dark energy flux ratio is larger for weaker solar wind driving. In the 1,400–1,600 MLT sector the integrated (peak) energy flux ratio is 1.87 and 1.99 for the NH and SH, respectively (see Fig. 4d). While the auroral energy flux is small at midday (e.g., the midday gap), its increase from darkness to sunlight is the largest among the three sectors. In the morning warm spot, the increase of auroral energy flux is smallest. This is probably because auroras in the dawn sector are mainly of the diffuse type. Diffuse auroras in the dawn sector are associated with precipitation of earthward and eastward drifting electrons from nightside central plasma sheet and are associated with nightside geomagnetic activity.

## Discussion

We have compared the response of auroral energy flux in the dayside part of the Northern and Southern Hemispheres to sunlight with FUV emission data acquired by TIMED/GUVI from 2002 to 2007 continuously. Our statistical result clearly shows significant enhancements in the dayside auroral energy flux from a dark to a sunlit ionosphere, as expected from previous studies of northern hemispheric auroras [e.g.,^9,10^]. The present study also demonstrates, for the first time, that the same auroral sunlight effect that was first discovered in the Northern Hemisphere also exists in the Southern Hemisphere and suggests an inter-hemispheric asymmetry in the dayside auroras due to the unequal sunlight received by the two hemispheres.

The sunlight enhancement of auroras was found to occur at all dayside local times. In the prenoon sector, the auroral energy flux increase is smaller. The diffuse type of electrons dominates particle precipitation in the dawn sector of the auroral oval^18^. Diffuse auroras show little sunlight effect^11^. In the noon sector, the auroral energy flux increase in sunlight is consistent with the fact that the cusp has much higher fluxes in summer hemisphere^11^. While the enhancement of auroral energy flux occurs at all dayside local times, the afternoon sector clearly stands out when the solar wind driving is and has been low for a few hours. The major source of particle precipitation in the postnoon sector around 1,500 MLT is monoenergetic events associated with the LLBL and BPS [e.g.,^18^]. Past studies have shown that monoenergetic auroral energy flux in this local time increase from winter to summer^11^, unlike their counterpart in the dusk-to-midnight quadrant, which is suppressed in sunlight^13^. Ion precipitation also contributes to FUV auroral emission in this local time sector. However, the global ion precipitation increase is less than ~ 4% from winter to summer^22^ and cannot account for the much larger increase observed in this study. Furthermore, ion precipitation contributes mostly to the nightside aurora and very little to the dayside aurora^18^. Therefore, we may rule out ion precipitation as the major cause.

Enhancements of the large-scale R-1/R-2 currents in the sunlit over the dark hemisphere have been reported previously^23^. These studies found an increase (by a factor of ~ 2 for geomagnetic quiet time periods) in the upward and downward R-1/R-2 FACs on dayside (0,800–1,800 MLT) and nearly unchanged FACs in the midnight-to-morning (0000–0,800 MLT) sector. The present finding of the auroral energy flux enhancement is consistent with the reported FAC enhancements. A later study also found that the current density of FACs at DMSP altitudes in the dusk-to-premidnight (16–22) sector tends to be larger in the illuminated than in the unilluminated events^24^. The cause of the increase of FACs in sunlight is still not well understood. Solar illumination is the primary cause of ionospheric ionization in the afternoon sector. Therefore, the finding that the R-1 FACs are linearly correlated with the ionospheric conductivity suggests a voltage generator in the dayside magnetosphere as the driver of the dayside R-1/R-2 FACs^25^. On the other hand, a higher Pedersen conductance in the sunlit ionosphere implies stronger coupling between plasma and neutrals and hence more inertia, and hence requires larger magnetic stress forces in the magnetosphere and in the ionosphere. Because the magnetic stress is released by the FAC, a larger ionospheric conductance would draw more FAC and create brighter auroras.

It is worth noting that the BPS, which is on closed field lines, is one of the major sources of the afternoon electron precipitation^1^, the auroral hot spots^3^, and upward R-1 FACs^26^. It is also known that the BPS is the major source of electron acceleration events (i.e., auroral arcs). On the nightside, monoenergetic acceleration events are suppressed in sunlight^5^. Explanation of the nightside auroral sunlight effect often invokes an ionospheric feedback theory^27^. The theory assumes an auroral generating circuit driven by a current generator in the nightside magnetosphere. In a dark ionosphere, the conductance is low. A potential drop is generated along the magnetic field and generates acceleration events to close the current loop. On the other hand, in a sunlit ionosphere, the conductance is high. When the conductance is high enough to support the current driven by the magnetosphere, field-aligned potential drops and electron acceleration events will not be generated. In the afternoon sector, electron precipitation from the BPS is expected to resemble the behavior of its nightside counterpart. Because the BPS is closer to the Earth than the LLBL, it maps to lower-latitude part of the auroral oval, equatorward of the lower-latitude boundary. We expect the auroral response to sunlight in the afternoon sector will have a mixed effect, meaning an energy flux decrease at lower latitudes and increase at higher latitudes in sunlight. The present study shows a larger (smaller) auroral energy flux increase from darkness to sunlight at higher (lower) latitude. We suspect that the LLBL voltage generator may be dominating in generating afternoon auroras and the averaging process may have smeared the result. We will address this issue in detail in a future work.

**Figure 5:**
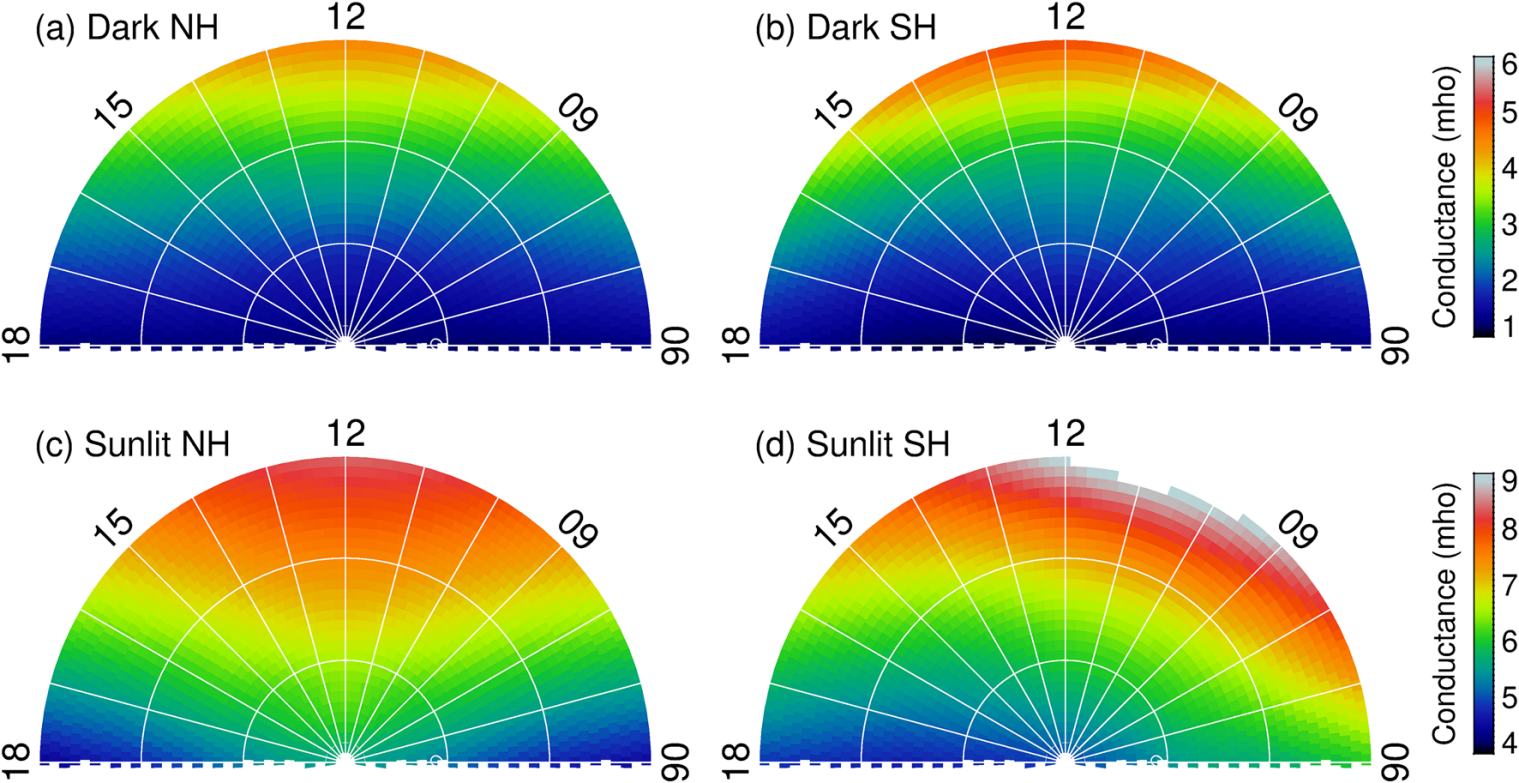
Dayside maps of the Pedersen photo-conductance for (**a**) dark NH, (**b**) dark SH, (**c**) sunlit NH, and (**d**) sunlit SH. The figure is produced by the first author using the Interactive Data Language (IDL) software version 8.2.3. The ULR link for IDL is https://www.harrisgeospatial.com/.

The dayside auroral energy flux in both hemispheres shows an increase from darkness to sunlight and the increase is larger in the Southern than in the Northern Hemisphere. This north–south asymmetry may be due to the north–south asymmetry in the Earth magnetic field. The larger tilt of the south magnetic dipole axis allows the dayside ionosphere to receive more sunlight, thus increasing the ionospheric conductance and field-aligned currents. In addition, there is an asymmetry in the magnitude and distribution of the magnetic field in the northern and southern polar region^28^. Both of these factors are expected to cause an asymmetry in the ionospheric conductivity in the polar region even if the solar radiation stays the same. It has been shown that the asymmetry in the north–south field and solar illumination can affect the occurrence of auroral substorms^29^. Therefore, it is reasonable to suspect that this north–south asymmetry can also cause a north–south asymmetry in the aurora energy flux. To explore this possibility, we calculate the ionospheric Pedersen conductance generated by photo-ionization using an empirical formula^10,30^. The result is shown in Fig. 5 for weak solar wind driving as shown in Fig. 2. Note that the result for all solar wind driving is similar and will not be shown and discussed here. The photo-conductance is calculated for bins where data are available, thus representing the ionospheric background conductance where electron precipitation occurred. When the ionosphere is in darkness (see Fig. 5a,b), there is a little difference in the Pedersen conductance between the two hemispheres, especially in regions where auroras typically occur. On the other hand, when the ionosphere is in sunlight (Fig. 5c,d), the distribution and magnitude of the Pedersen conductance show a significant difference between the two hemispheres. For example, in the 1,400–1,600 MLT sector, the mean photo-conductance between 72° and 80° MLat is 6.43 mho in the NH and 5.82 mho in the SH. The north–south percentage difference is ~ 1% for a dark ionosphere and ~ 10% for a sunlit ionosphere. The small (~ 1%) difference in the ionospheric photo-conductance implies that contribution of the difference in the magnetic field magnitude to the conductance is small and negligible. If the postnoon aurora is generated mainly by a voltage generator in the LLBL as described above, one would expect that the NH will have a larger auroral increase than the SH. This is consistent with the present study result for regions poleward of the energy flux peak. The region equatorward of the energy flux peak is likely associated with BPS precipitation and its auroral response to sunlight is controlled by the ionospheric feedback mechanism, meaning suppression of the aurora in sunlight.

Finally, it is worth pointing out that a larger (~ 6–20%) sunlight-to-darkness auroral energy flux increase in the SH than in the NH may be associated with a larger ionospheric conductivity in the SH. Indeed, the SH receives slightly more insolation when integrated over the year. This is because the orbit of the Earth around the Sun is not circular but elliptical and because the Earth is closer to the Sun during northern winter (southern summer) by a few (~ 3.4%) percent. The distance between the Sun and the Earth is 1.52 × 10^8^ km at aphelion and 1.47 × 10^8^ km at perihelion. Since the solar radiation is inversely proportional to the square of the distance from the Sun, the difference between the Sun and the Earth can result in ~ 7% more solar radiation at the Earth’s orbit during northern winter than during northern summer. Note that this effect is not included in the above empirical Pedersen conductance model, although it is not likely to offset the north–south conductance difference resulting from solar insolation and magnetic field asymmetry.

## Conclusions

We have investigated the response of dayside auroras to sunlight using the FUV emission data acquired by the GUVI imaging spectrometer on board the TIMED spacecraft from 2002 to 2007. We compared auroral energy flux deposited into the sunlit and dark hemispheres for both Northern and Southern Hemispheres. It was found enhanced dayside auroras in the sunlit hemisphere for both hemispheres. The increase in the auroral energy flux was significant (by a factor of up to ~ 2) over the dayside sector. The present result is consistent with previous findings that large-scale dayside field-aligned currents increase in summer against the winter season and suggests a voltage driven dayside magnetosphere-ionosphere system. Although there is a complexity in the role of the background ionospheric conductance that plays in the dayside aurora, the present result suggests that the dayside aurora is rarely symmetric probably because of the north–south asymmetry in the ionospheric photo-conductance.
